# Deep learning for spirometry quality assurance with spirometric indices and curves

**DOI:** 10.1186/s12931-022-02014-9

**Published:** 2022-04-21

**Authors:** Yimin Wang, Yicong Li, Wenya Chen, Changzheng Zhang, Lijuan Liang, Ruibo Huang, Jianling Liang, Dandan Tu, Yi Gao, Jinping Zheng, Nanshan Zhong

**Affiliations:** 1 National Center for Respiratory Medicine, State Key Laboratory of Respiratory Disease, National Clinical Research Center for Respiratory Disease, Guangzhou Institute of Respiratory Health, First Affiliated Hospital of Guangzhou Medical University, Yanjiang Road 151, Guangzhou, 510120 Guangdong People’s Republic of China; 2 Tsinghua-Berkeley Shenzhen Institute, Tsinghua University, Shenzhen, 518055 China; 3 Huawei Cloud BU EI Innovation Laboratory, Huawei Technologies, Shenzhen, 518129 China

**Keywords:** Artificial intelligence, Deep learning, General practitioner, Quality control, Spirometry

## Abstract

**Background:**

Spirometry quality assurance is a challenging task across levels of healthcare tiers, especially in primary care. Deep learning may serve as a support tool for enhancing spirometry quality. We aimed to develop a high accuracy and sensitive deep learning-based model aiming at assisting high-quality spirometry assurance.

**Methods:**

Spirometry PDF files retrieved from one hospital between October 2017 and October 2020 were labeled according to ATS/ERS 2019 criteria and divided into training and internal test sets. Additional files from three hospitals were used for external testing. A deep learning-based model was constructed and assessed to determine acceptability, usability, and quality rating for FEV_1_ and FVC. System warning messages and patient instructions were also generated for general practitioners (GPs).

**Results:**

A total of 16,502 files were labeled. Of these, 4592 curves were assigned to the internal test set, the remaining constituted the training set. In the internal test set, the model generated 95.1%, 92.4%, and 94.3% accuracy for FEV_1_ acceptability, usability, and rating. The accuracy for FVC acceptability, usability, and rating were 93.6%, 94.3%, and 92.2%. With the assistance of the model, the performance of GPs in terms of monthly percentages of good quality (A, B, or C grades) tests for FEV_1_ and FVC was higher by ~ 21% and ~ 36%, respectively.

**Conclusion:**

The proposed model assisted GPs in spirometry quality assurance, resulting in enhancing the performance of GPs in quality control of spirometry.

**Supplementary Information:**

The online version contains supplementary material available at 10.1186/s12931-022-02014-9.

## Introduction

Spirometry is the most frequently used pulmonary function test (PFT) in the assessment of lung function to provide information used in the diagnosis, screening, and monitoring of respiratory diseases [1]. Over the past decades, spirometry has become easy access across levels of healthcare tiers even in communities thanks to the availability of portable spirometers and the training of operators [2]. High-quality assurance of spirometry testing is pivotal for the proper management of patients with respiratory diseases [3], as well as in researches where spirometry is often needed for drug qualification trials and epidemiological surveys.

In 2019, the American Thoracic Society (ATS) and the European Respiratory Society (ERS) jointly updated technical standards for conducting spirometry [4]. The updated document contains quantitative and visual inspection criteria, moreover, recommendations on the acceptability, usability, and rating for FEV_1_ and FVC maneuvers are also provided [4]. A motivated and trained operator is a key element to obtain high-quality spirometry data since only the trained operators can complete the visual assessment of spirometry quality control. The quality of spirometry performed in primary care practice can differ on the impact of formal training [5, 6]. In China, only 12.0% of patients with chronic obstructive pulmonary disease reported the previous PFT [7], which is partly due to the shortness of skilled and trained operators. The government had promoted 50% of the primary care settings to be equipped with spirometers, which could output lung function parameters, flow–volume curves, and volume–time curves. What’s more, the utilization rate of spirometers in primary care settings should reach 90%, which were performed by general practitioners (GPs). These measures have led to the need for GPs to have powerful supporting tools to assist in the quality control of spirometry.

Several studies have already incorporated information technologies into the automatic process of spirometry quality assurance to help non-specialist operators in clinical practice. Researchers elaborated and validated the performance of a Clinical Decision Support System for spirometry quality assessment [8]. Andrew and colleagues [9] used conventional machine learning algorithms to classify four types of user errors in spirometry, namely, early termination, cough, variable flow, and extra breath. Similarly, several machine learning classifiers were also utilized to decide the curves’ acceptability in spirometry [10]. More recently, authors leveraged deep learning techniques, i.e., convolutional neural network (CNN) to classify FEV_1_ and FVC acceptability and usability in spirometry [11]. Since deep learning models have a large parameter space and have automatically learned features. Deep learning-based computer vision (CV) methods outperform those traditional CV in most tasks [12–16]. In addition, updated standards for spirometry have led to the need for a more progressive approach for spirometry quality to meet the updated criteria.

Comparing to previous literature, we aimed to develop a more advanced approach in this work by combining the ATS/ERS 2019 guidelines with an object detection module, which not only can provide acceptability, usability, and rating assessment for FEV_1_ and FVC separately with state-of-the-art accuracy but are also able to classify and locate common artifacts visually.

## Materials and methods

The present study was approved by the Ethics Committee of the First Affiliated Hospital of Guangzhou Medical University (approval number: 2020124). Since it was an anonymized and retrospective research, written informed consent was waived.

### Spirometry

Spirometry was performed by technicians following ATS/ERS 2005 guidelines [17] and Chinese Thoracic Society 2014 guidelines [18]. Tests using for training and testing the model were acquired with MasterScreen-Pneumo (Jaeger, Germany) equipment. In primary care units, tests were performed by portable spirometer with U-BREATH PF680 (E-linkcare, China).

### Study design

In accordance with ATS/ERS 2019 standards [4], errors of all flow–volume and volume–time curves that need visual inspection were labeled independently by four pulmonologists, whose experience in operating and interpreting spirometry for more than 2 years. They labeled different curves (never the same). If there were any doubt, then the expert (YG) the experience of more than 18 years would make the final decision. The quantitative criteria were checked by the Python scripts based on the rules of standards [4]. The details of quantitative and visual criteria are shown in Additional file 1: Tables S1 and S2. A cloud-based artificial intelligence (AI) system was constructed based on the established deep learning model (Additional file 1: Appendix S1). The architecture, input, and output of the system are shown in Additional file 2: Fig. S1, Additional file 3: Fig. S2, and Additional file 4: Fig S3.

Following a 3-month period, the baseline performance of GPs who have received spirometry training was assessed in month 0 before the intervention. GPs were instructed to manage the AI system after baseline data were collected, they were able to access the system during the period of the first and second intervention months (month 1 and month 2). The method procedure was illustrated in Additional file 5: Fig S4. The system was introduced to GPs to assist their daily practice, to evaluate the efficacy of this new approach of working. The research was conducted from March 2021 to May 2021 in ten selected primary care units in Guangzhou China. A total of 30 GPs was recruited, they were trained to perform the spirometry according to ATS/ERS 2019 standards [4]. Patients were selected for the study who required spirometry testing according to GPs’ decision, without limitation of age or sex. All tests performed were selected to assess GPs’ performance. The patients’ names, technicians’ names, and physicians’ names were all anonymized. GPs could only access a patient’s data, the one who performed spirometry under their own guidance in their routine work, they could not access other patients’ spirometry data.

### Data acquisition

Inclusion criteria included the spirometry files with at least one usually three flow–volume and volume–time curves, regardless of age, pattern, and quality features. A typical example of a spirometry file is shown in Additional file 3: Fig S2. Files with curves that could not be resolved to a JSON (i.e. The flow–volume and volume–time curves were resolved to numeric values over a 0.01-s interval during the maneuver) file were excluded. The training set and internal test set files were consecutively retrieved from the PFT databases of the First Affiliated Hospital of Guangzhou Medical University between October 2017 and October 2020. Files were randomly divided into 90% for training and 10% for internal testing.

The external test files were retrieved from the PFT database of the National Clinical Research Center for Respiratory Disease between January 2021 and February 2021.

### Model development

**Fig. 1:**
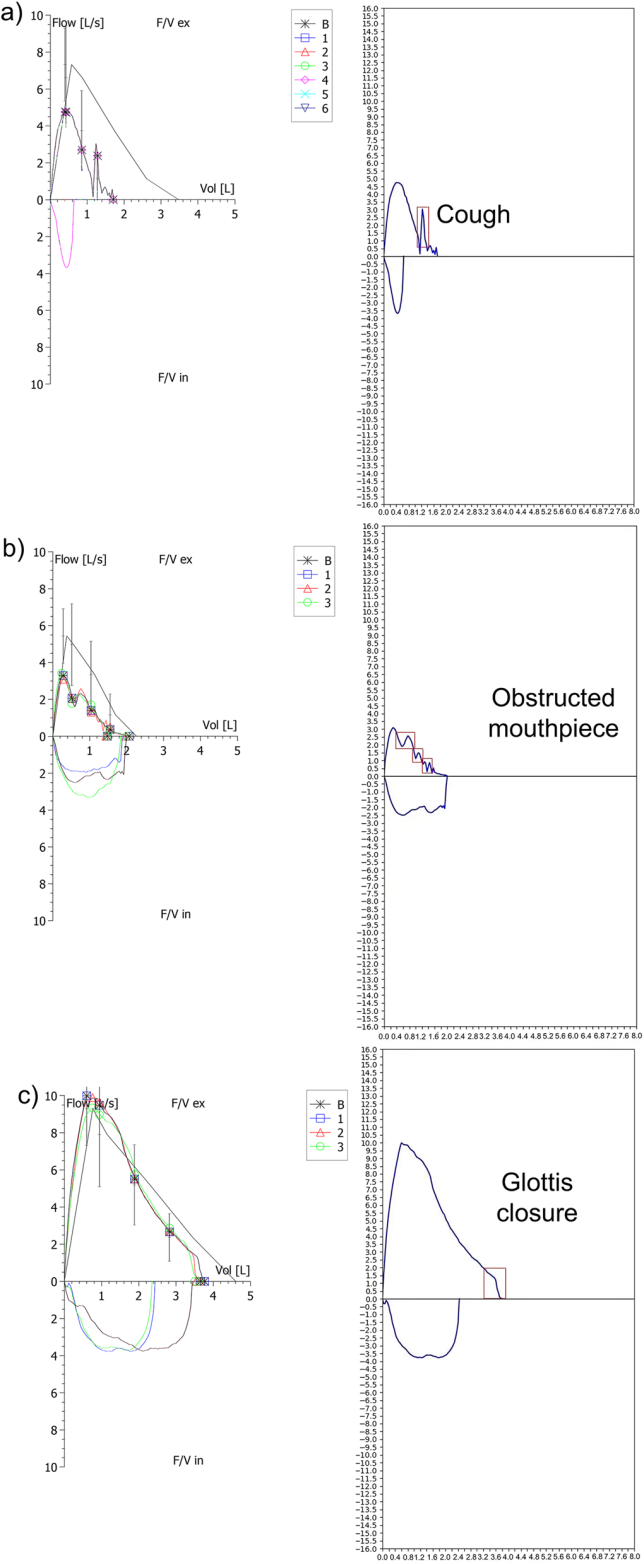
Input and output samples from Object Detection Module. **a** Suspect a cough in the flow–volume graph, an up-and-down flow spike is detected by the module; **b** Suspect obstructed mouthpiece or spirometer in the flow–volume graph, a flutter is detected by the module; **c** Suspect glottis closure in the flow–volume graph, a sharp drop is detected by the module

As shown in Additional file 6: Fig S5, our framework mainly consisted of three modules: Data Preprocessing Module, Rule Module, and Object Detection Module. Given input data, the Data Preprocessing Module would extract both numerical information (lung function parameters, flow–volume curve data, and volume–time curve data) and curve images. Then, the Rule Module would process the numerical information and output corresponding results (Yes/No). Simultaneously, the flow–volume and volume–time curve images were sent to the Object Detection Module, which would automatically determine the type and location if an anomaly exists (examples of input and output images were shown in Fig. 1). The Module had a popular ResNet50 as the backbone network for feature extraction. Details of the ResNet50-V1 architecture were shown in Additional file 7: Fig. S6. Finally, the results of the Rule Module and Object Detection Module were combined together to generate FEV_1_ or FVC acceptability, usability, quality rating, and guidance for patients (Additional file 1: Table S3). All the data we used would not be furtherly saved inside the two modules once the results were generated. Details on the model development can be found in Additional file 1: Appendix S2.

### Statistical analysis

The proposed algorithm was evaluated on both the internal and external test sets. Five metrics including balanced accuracy, sensitivity, specificity, PPV, and NPV were utilized for evaluation of FEV_1_ and FVC acceptability and usability. Overall accuracy is utilized for the evaluation of quality ratings. Comparisons among the monthly percentages of acceptable, usable maneuvers, and good quality tests for FEV_1_ and FVC, in month 0, month 1, and month 2 were carried out using the chi-squared test. Statistical analyses were performed with SPSS version 26 (Statistical Package for Social Science) and Python.

## Results

### Participant characteristics

**Fig. 2:**
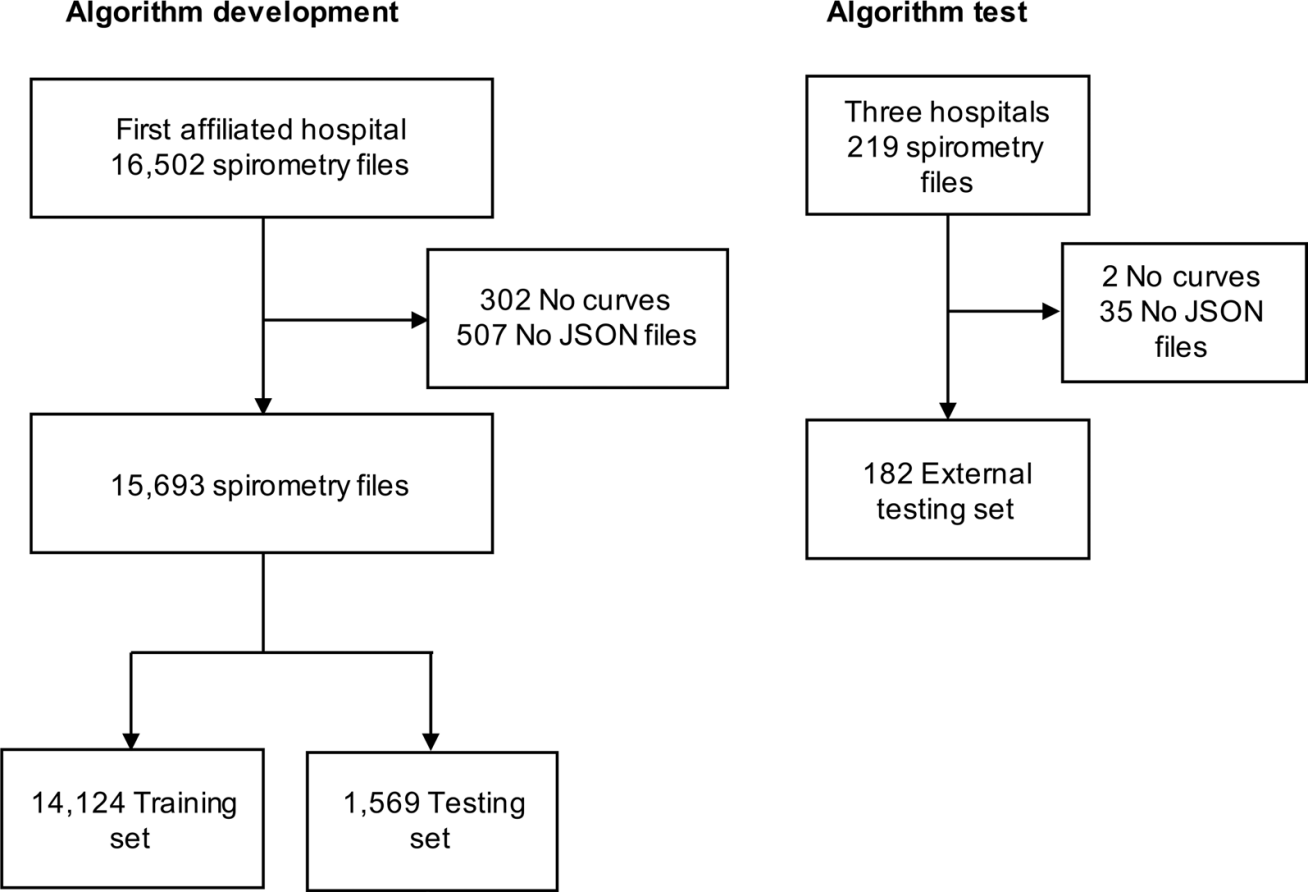
Flowchart of the algorithm training and testing data acquisition, selection, and division. 16,502 spirometry files were retrieved from the First Affiliated Hospital of Guangzhou Medical University. After exclusion of files with no curves or curves that could not be resolved, 15,693 files remained, files were randomly divided into training and internal testing sets. Additional 219 spirometry files retrieved from the multicenter (three hospitals) of the National Clinical Research Center for Respiratory Disease were used for external testing. *FEV*_*1*_  forced expiratory volume in 1 s, *FVC* forced vital capacity

A total of 16,502 files were scanned and labeled. After the exclusion of files with no curves or curves that could not be resolved (n = 809), 15,693 files remained, the training set involved 14,124 files. In the internal test set, there were 4592 curves from 1569 files. The external test set involved an extra 360 curves from 182 files. The flowchart of data acquisition, selection, and division is presented in Fig. 2. Details on the types of abnormalities that were identified can be found in Additional file 1: Table S4.

**Fig. 3:**
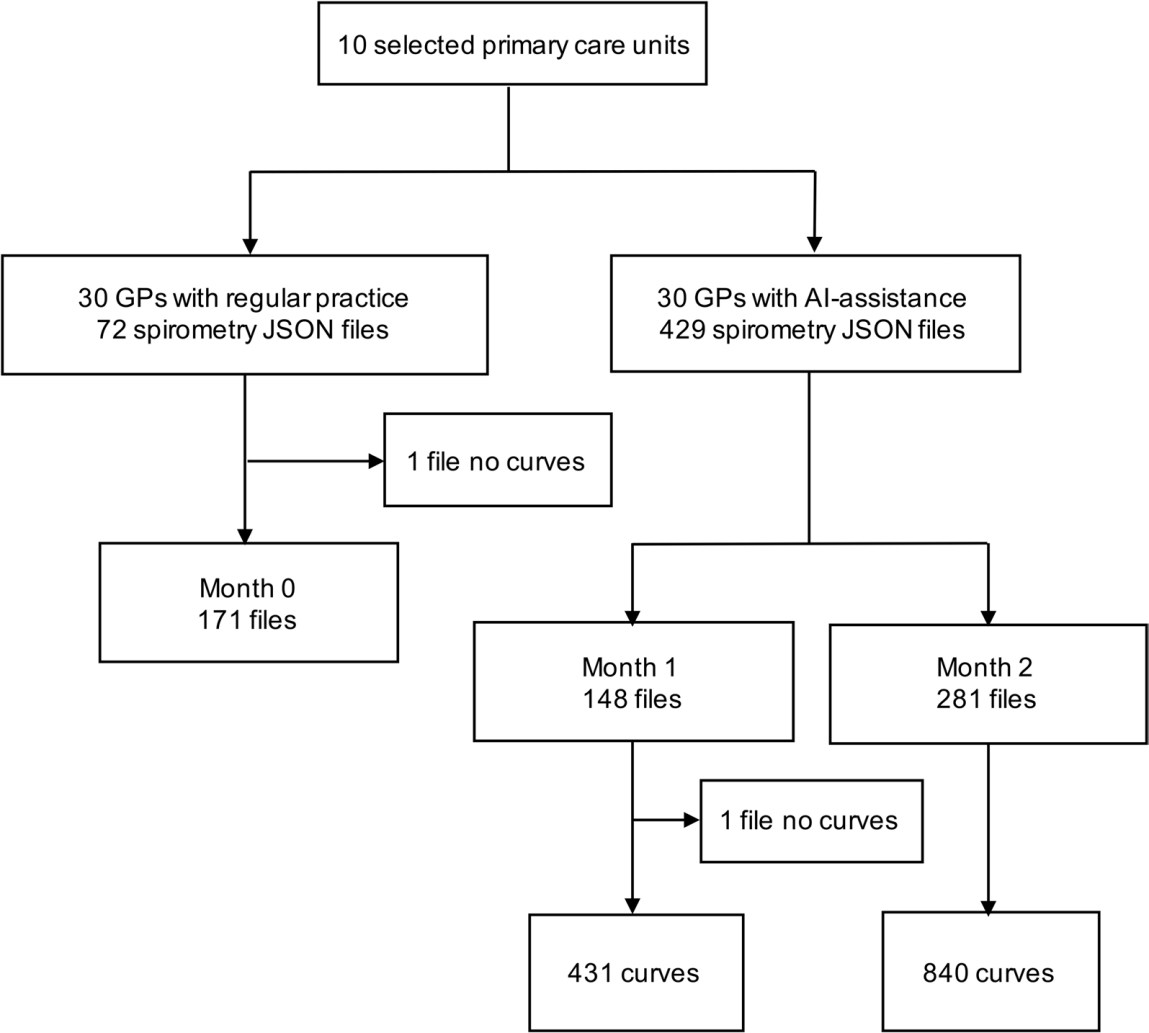
Flowchart of data acquisition from ten primary care units. 171, 431, and 840 curves from 72, 148, and 281 files performed during month 0, month 1, and month 2 in ten primary care units by 30 GPs, respectively. *GPs* general practitioners; *AI* artificial intelligence

At the end of the 3-month study, 501 files had been performed from ten primary care centers. After the exclusion of files with no curves (n = 2), there were 171, 431, and 840 curves from 72, 148, and 281 files performed during month 0, month 1, and month 2, respectively. Flowchart of data acquisition was shown in Fig. 3. We observed that the monthly number of tests showed an astonishing increasing trend from the beginning to month 2.

### Model testing

We first evaluated the proposed model regarding FEV_1_ and FVC acceptability, usability, and quality rating in the internal test set (n = 4592 curves). It can be observed that our model achieved promising results in all three tasks. The model prediction of FEV_1_ acceptability and usability resulted in promising balanced accuracy of 95.1% and 92.4% with high sensitivity (97.8% and 99.4%) but low NPV (69.6% and 72.2%), respectively. The model prediction of FVC acceptability and usability also achieved well-balanced accuracy (93.6% and 94.3%) (Table 1). We also observed excellent accuracy in both FEV_1_ and FVC ratings (94.3% and 92.2%, respectively, Table 2). Detailed statistical data in the internal test set is shown in Additional file 8: Fig. S7 and Additional file 9: Fig. S8.

**Table 1:** Acceptability and usability assessment in the internal and external test set (n = 4592 and 360 curves, respectively)

Task	Balanced accuracy (%)	Sensitivity (%)	Specificity (%)	PPV (%)	NPV (%)
*Internal test set*					
FEV_1_ Acceptability	95.1	97.8	92.4	99.6	69.6
FEV_1_ Usability	92.4	99.4	85.4	99.7	72.2
FVC Acceptability	93.6	97.5	89.6	98.9	79.4
FVC Usability	94.3	99.5	89.0	99.8	74.7
*External test set*					
FEV_1_ Acceptability	97.7	99.6	95.8	98.0	99.1
FEV_1_ Usability	100.0	100.0	100.0	100.0	100.0
FVC Acceptability	95.4	99.6	91.3	94.9	99.2
FVC Usability	100.0	100.0	100.0	100.0	100.0

*FEV*_*1*_ forced expiratory volume in 1 s, *FVC* forced vital capacity, *PPV* positive predictive value, *NPV* negative predictive value

**Table 2:** Rating assessment in the internal and external test set (n = 1569 and 182 files, respectively)

Task	Accuracy (%)
*Internal test set*	
FEV_1_ quality rating	94.3
FVC quality rating	92.2
*External test set*	
FEV_1_ quality rating	95.6
FVC quality rating	92.3

See Table 1 legends for abbreviations

Next, the performance of our model was evaluated on the external test set to validate its generalization ability. As shown in Table 1, our model expressed astonishing performance with a 100% accuracy of FEV_1_ and FVC usability specifically. This phenomenon may be due to the relatively small size of the test set but indeed validate the strong generalization ability of the proposed model to some extent. The model to determine acceptability criteria also demonstrated excellent accuracy (details in Table 1). The overall accuracy of FEV_1_ grade and FVC grade were 95.6% and 92.3%, respectively (Table 2). Detailed statistical classification in the external test set is shown in Additional file 9: Fig. S8 and Additional file 10: Fig. S9.

### Effects of the AI system on quality

The monthly percentages of acceptability and usability maneuvers were significantly and consistently higher in the AI-assistance groups (month 1 and month 2) than in the baseline group (month 0) (Table 3). In month 2, with the AI supporting, averages of 91.8% for FEV_1_ and 89.4% for FVC acceptability maneuvers were presented. In the contrast, the baseline showed lower mean percentages (81.9% and 70.2%, respectively) (both* P* < 0.0001). The percentages of FEV_1_ and FVC usability maneuvers maintained the positive trend (88.3% and 88.9% at month 0, 99.2% and 99.2% at month 2) (both *P* < 0.0001, Table 3).

**Table 3:** FEV_1_ and FVC quality assessment in primary care units

	GPs with regular practice	GPs with AI-assistance		
Task, n (%)	Month 0	Month 1	Month 2	*P* value
*Acceptable*	*N = 171 curves*	*N = 431 curves*	*N = 840 curves*	
FEV_1_ maneuvers	140 (81.9%)	359 (83.3%)	771 (91.8%)	< .0001
FVC maneuvers	120 (70.2%)	343 (79.6%)	751 (89.4%)	< .0001
* Usable (including acceptable)*	*N = 171 curves*	*N = 431 curves*	*N = 840 curves*	
FEV_1_ maneuvers	151 (88.3%)	396 (91.9%)	833 (99.2%)	< .0001
FVC maneuvers	152 (88.9%)	398 (92.3%)	833 (99.2%)	< .0001
*Good quality ratings (A, B, or C grades)*	*N = 72 files*	*N = 148 files*	*N = 281 files*	
FEV_1_ tests	51 (70.8%)	117 (79.1%)	258 (91.8%)	< .0001
FVC tests	38 (52.8%)	107 (72.3%)	250 (89.0%)	< .0001

Data are presented as absolute numbers in the case of frequencies. *GPs*  general practitioners, *AI*  artificial intelligence; see Table 1 legends for expansion of abbreviations

The higher monthly percentages of good quality ratings (A, B, or C grades) for FEV_1_ and FVC in month 1 (79.1% and 72.3%) and month 2 (91.8% and 89.0%) were obtained in comparison with month 0 (70.8% and 52.8%), respectively (both *P* < 0.0001) (Table 3).

## Discussion

The current research has presented a novel framework combining both advanced object detection algorithm and ATS/ERS 2019 standards to enable automatic spirometry quality assurance, specifically, determination of acceptability, usability, and quality rating for FEV_1_ and FVC. The object detection module learned domain knowledge during training and was able to mimic the visual screening action of clinical experts during inference. Extensive experiments show that our proposed framework could achieve excellent performance on internal testing as well as external testing. Furthermore, the AI system enhanced the quality of tests covering acceptability, usability, and good quality ratings (A, B, or C grades) for FEV_1_ and FVC performed by GPs. Consequently, the results seem to demonstrate that our approach could help GPs who were not specialized professionals to carry out spirometry testing with high quality.

Quality control of spirometry testing with information and communications technology is not a new idea, and it has been shown that such approaches do improve the performances of clinicians in primary care [19]. Nowadays, we regularly use them to interpret chest computed tomography, or PFT, to analyze lung irregularities, or as indicators for respiratory disease diagnosis and exacerbation [20–24]. Although automated assessment quality of spirometry had been evaluated before [11, 25], none has become a clinical reality covering multiple regions around the world. Firstly, there is marked difficulty in assaying data from various spirometers. Secondly, there are a lot of guidelines that can be selected to assess spirometry quality [4, 17, 18, 26], which may cause controversy and puzzles for non-specialized clinicians. Different recommendations on spirometry quality control to use will influence the interpretation of spirometry tests. The usefulness and strength of our approach lie in the ability to meet the updated criteria in order to increase the accuracy, precision, and quality of spirometric measurements.

Leveraging the deep learning-based model, we approached FEV_1_ and FVC acceptability and usability as having both quantitative criteria and visual criteria on the spirometry. As such, the deep learning model is able to detect subtle characteristics and artifacts that are challenging for humans to identifies and incorporates into a powerful quality assurance model. More concretely, the model can learn high-level features corresponding to different artifacts by training on a large number of known quality control cases, then it can generate the type of the anomaly and its position during the test phase given input data, a process that is similar to how students learn new knowledge. The most recent work proposed by Das et al. [11] formulated this quality assurance task as a classification problem and tackled it by using a CNN model. However, we argued that this method could only classify the acceptability and usability of spirometry curves. On the contrary, the object detection module we used could not only classify the image type but also locate the position of the abnormalities simultaneously. This feature could serve as interpretable guidance for the GPs. In addition, our approach could provide explicit feedback to the operator. Warning messages and suggested corrections are also remotely provided to the operator in real-time online, thereby, the operator in primary care could decide whether there’s a need to do an additional maneuver. Furthermore, the departments need to undertake regional spirometry quality assessment, then they can use our model for retrospective evaluation spirometry from communities under its management.

In our study, the most common visual criteria that could not be achieved by the human experts were glottic closure and cough although they have a visual inspection of flow–volume and volume–time curves. Therefore, our model was focus on dealing with these visual artifacts better in line with clinical practice. Another advantage of this research was that the algorithms could integrate into the spirometry system software of various equipment.

The poor performance of GPs in primary care on quality assurance of spirometry using the baseline data was observed in this study, which is in accordance with previous studies [6, 27, 28]. The baseline data confirmed the need for assistance on quality control of the tests at the primary care level. Our data demonstrated that the AI system increases good quality (A + B + C grades) tests for FEV_1_ and FVC by ~ 21% and ~ 36% from month 0 to month 2. The results generated a beneficial impact on spirometry quality assurance.

A limitation of the current study was that the test sample we used may not entirely reflect the prevalence of quality artifacts of spirometry tests that operators in primary care confront in daily practice. We did not explore the criterion of the leak since we only labeled two files, one reason is that a leak rarely occurred in skilled technicians. But with the application of this model in primary care, this criterion will be incorporated. Additionally, we did not test the level of agreement of authors when labeled the quality anomaly. Furthermore, the time to evaluate the performance of the model to help GPs was short, which may limit sustained assessment of the impact of the AI system. Future longer time and additional centers will be included.

## Conclusions

To conclude, we developed a high-precision deep learning-based model for automated quality control of spirometry. The AI system established on this model could provide strong assistance for improving the GPs’ performance. It can be used with high scalability across the healthcare tiers.

## Supplementary Information

**Additional file 1: Appendix S1. **Description of the cloud-based AI system. Appendix S2-Model development.** Table S1.** Visual and quantitative criteria for FEV_1_ and FVC acceptability and usability according to ATS/ERS 2019 standardization. **Table S2. **Quality rating for FEV_1_ and FVC according to ATS/ERS 2019 standardization. **Table S3. **Warning trigger and guidance for patient according to ATS/ERS 2019 standardization. **Table S4. **Types of abnormalities and their respective prevalence.

**Additional file 2: Fig. S1.** Organizational structure of the cloud-based AI system.

**Additional file 3: Fig. S2.** Example of the patient file with a complete spirometry test.

**Additional file 4: Fig. S3.** AI system used to evaluate each patient case.

**Additional file 5: Fig. S4.** Method procedure.

**Additional file 6: Fig. S5.** Proposed framework and Object Detection Module.

**Additional file 7: Fig. S6.** Architecture of ResNet50-V1 backbone model.

**Additional file 8: Fig. S7.** Confusion matrices of FEV_1_ and FVC acceptability and usability in the internal test set. 0: Not acceptable/usable, 1: Acceptable/usable.

**Additional file 9: Fig. S8.** Confusion matrices of FEV_1_ and FVC quality rating (Internal/external).

**Additional file 10: Fig. S9.** Confusion matrices of FEV_1_ and FVC acceptability and usability in the external test set. 0: Not acceptable/usable, 1: Acceptable/usable.

## Data Availability

The dataset supporting the conclusions of this article is included within the article.

## Abbreviations

AI: Artificial intelligence
ATS/ERS: American Thoracic Society/European Respiratory Society
CNN: Convolutional neural network
FEV_1_: Forced expiratory volume in 1 s
FVC: Forced vital capacity
GP: General practitioner
PFT: Pulmonary function test
