# Supplementary material for: Deep learning for spirometry quality assurance with spirometric indices and curves
Source: Respir Res. 2022 Apr 21;23:98. doi: 10.1186/s12931-022-02014-9 (PMC9028127; doi:10.1186/s12931-022-02014-9)
Supplement: Supplementary file 1 — Additional file 1: Appendix S1. Description of the cloud-based AI system. Appendix S2-Model development. Table S1. Visual and quantitative criteria for FEV1 and FVC acceptability and usability according to ATS/ERS 2019 standardization. Table S2. Quality rating for FEV1 and FVC according to ATS/ERS 2019 standardization. Table S3. Warning trigger and guidance for patient according to ATS/ERS 2019 standardization. Table S4. Types of abnormalities and their respective prevalence. [file 12931_2022_2014_MOESM1_ESM.docx]

**Additional Material**

**Appendix S1**-**Description of the cloud-based AI system**

The AI system allowed the remote log-in using passwords for unlimited units. It was compatible with portable spirometers that could export the parameter and curve data in a JSON format (i.e. The flow-volume and volume-time curves were resolved to numeric values over a 0.01-second interval during the maneuver). Once the data had been performed, it could be uploaded and sent to the system within 10 min, on the one hand, an Oracle driver was used for the database, on the other hand, the AI algorithms were utilized to provide the interpretation and quality assessment of spirometry within 30 s. The coordinator was responsible for integrating data from primary care units into the system, together with sending feedback information to general practitioners (GPs). It ran across a network of nodes, each linking to a unit that could simultaneously access the system independently and has several spirometers that could be operated by different technicians (**Figure S1**).

The system had three main functionalities: 1) it displayed spirometer outputs using a PDF format, a representative example of a patient file is shown in **Figure S2**; 2) used AI algorithms to quality assessment of tests in accordance with guidelines[1]. The system assessed the quality of spirometry tests as the object, with each maneuver being decided for FEV_1_ and FVC (decide separately) acceptability and usability, together with one of seven grades (**Figure S3**). If FEV_1_ and/or FVC were not acceptable/usable, then the warning messages and patient instructions would be described. Tests were evaluated as good quality rating if they obtain a grade of A or B or C. 3) returned the results back to the GPs.

**Appendix S2**-**Model development**

**T**he Data Preprocessing Module would extract curve images. To be specific, flow-volume curves were exported from spirometers in JSON format and then reconstructed as curve images. All extracted curves were further processed to have a size of 600 ∗ 1200 pixels by using the OpenCV-Python package. The x-axis represented the volume whose range was 0 to +8 L with a minimum scale value of 0.2 L. The y-axis represented flow whose range was -16 to +16 L/s with a minimum scale value of 0.5 L/s. Note that the proposed model was capable of process curves of arbitrary sizes which gave us additional flexibility. Then we used an open-source tool called LabelImg[2] to annotate the curve images. Annotations included the coordinates (x-min, y-min, x-max, y-max) and the type of the anomaly regions in the curve images.

Particularly, the Object Detection Module could simultaneously realize anomaly classification task and position coordinates regression task given an input curve image[3] (part of the codes are based on <https://github.com/tensorflow/models/tree/master/research/object_detection>). The Module had a Faster R-CNN[4] alike architecture with the popular ResNet50[5] as the backbone network for feature extraction. Details of the ResNet50-V1 architecture were shown in **Figure S6**. The network has 16 bottlenecks covering 48 convolution layers in total, whose weights can be learned during the model training process. The most important building block of this ResNet50-V1 model is the bottleneck. It can be seen as a unit architecture including 3 convolution layers and a stack of the bottlenecks build the whole network. The skip connection [5] (solid curves and dotted curves) outside the bottleneck can help to train this very deep network and prevent the problem of gradient vanish. An input image will be processed through the whole network to generate a feature map. As can be seen in **Figure S5**, the feature map was extracted from an input image by ResNet50 convolutional neural network firstly. Next, a region proposal network would generate proposals, i.e., potential positions where a certain type of anomaly might exist according to the aforementioned feature map. Then for each of the proposals, a feature vector would be extracted from the feature map by the region of interest pooling layer. Finally, fully connected layers (include both classifier and regressor) received the feature vector and outputted the detected anomaly type and position.

As a common practice, we borrowed the multi-class loss from Fast R-CNN[6], which consisted of a classification loss and a regression loss for calculating the error between predictions and ground truth values. This Module was trained for a total of 30,000 iterations, with a learning rate of 1e-3 for 20,000 iterations and followed by 10,000 iterations with a learning rate of 1e-4. We used a stochastic gradient descent optimizer to train the model and set the batch size to 1 through all iterations. The whole framework was developed using Python and the Object Detection Module was implemented and trained using TensorFlow 1.14.0 on 4 Tesla K80 GPUs.

**References**

1. Graham BL, Steenbruggen I, Miller MR, Barjaktarevic IZ, Cooper BG, Hall GL, et al. Standardization of Spirometry 2019 Update. An Official American Thoracic Society and European Respiratory Society Technical Statement. Am J Respir Crit Care Med. 2019;200(8):e70-e88.

2. Tzutalin. Git code (2015). <https://github.com/tzutalin/labelImg>.

3. Huang J, Rathod V, Sun C, Zhu M, Korattikara A, Fathi A, et al. Speed/Accuracy Trade-Offs for Modern Convolutional Object Detectors. In 2017 IEEE Conference on Computer Vision and Pattern Recognition (CVPR); 21-26 July 2017. 2017: 3296-3297.

4. Ren S, He K, Girshick R, Sun J. Faster R-CNN: Towards Real-Time Object Detection with Region Proposal Networks. IEEE T Pattern Anal. 2017;39(6):1137-1149.

5. He K, Zhang X, Ren S, Sun J. Deep Residual Learning for Image Recognition. In 2016 IEEE Conference on Computer Vision and Pattern Recognition (CVPR); 27-30 June 2016. 2016: 770-778.

6. Girshick R. Fast R-CNN. In 2015 IEEE International Conference on Computer Vision (ICCV); 7-13 Dec. 2015. 2015: 1440-1448.

**Table S1** Visual and quantitative criteria for FEV_1_ and FVC acceptability and usability according to ATS/ERS 2019 standardization[1]

|  | **Acceptability** | |  | **Usability** | |
| --- | --- | --- | --- | --- | --- |
| **Criterion** | **FEV_1_** | **FVC** |  | **FEV_1_** | **FVC** |
| **Visual criteria** |  |  |  |  |  |
| No cough in the first 1 s of expiration | Required | Not required |  | Required | Not required |
| No glottic closure in the first 1 s of expiration | Required | Required |  | Required | Required |
| No glottic closure after 1 s of expiration | Not required | Required |  | Not required | Not required |
| No obstructed mouthpiece or spirometer | Required | Required |  | Not required | Not required |
| No leak | Required | Required |  | Not required | Not required |
| **Quantitative criteria** |  |  |  |  |  |
| BEV ≤5% of FVC or 0.100 L,  whichever is greater | Required | Required |  | Required | Required |
| Achieve one of the EOFE indicators:  1. Time of end FVC ≥15 s  2. Expiratory plateau (≤0.025 L in the last 1 s of expiration)  3. FVC is within the repeatability tolerance of or is greater than the largest prior observed FVC | Not required | Required |  | Not required | Not required |
| Rise time ≥150 ms | Required | Required |  | Not required | Not required |
| FIVC – FVC |  |  |  |  |  |

Abbreviations: BEV = back-extrapolated volume; EOFE = end of the forced expiration; FEV_1_ = forced expiratory volume in 1s; FIVC = forced inspiratory vital capacity; FVC = forced vital capacity.

**Table S2** Quality rating for FEV_1_ and FVC according to ATS/ERS 2019 standardization[1]

| **Ratings** | **Number of Measurements** | **Repeatability: Age** >6 yr |
| --- | --- | --- |
| **A** | ≥3 acceptable | Within 0.150 L |
| **B** | 2 acceptable | Within 0.150 L |
| **C** | ≥2 acceptable | Within 0.200 L |
| **D** | ≥2 acceptable | Within 0.250 L |
| **E** | ≥2 acceptable or 1 acceptable | >0.250 L |
| **U** | 0 acceptable and ≥1 usable | Not applicable |
| **F** | 0 acceptable and 0 usable | Not applicable |

Abbreviations see Table S1 legends.

**Table S3** Warning trigger and guidance for patient according to ATS/ERS 2019 standardization[1]

| **Warning trigger** | **Guidance for patient** |
| --- | --- |
| BEV exceeds limit | Blast out immediately when completely full |
| Rise time ≥150 ms | Blast out immediately when completely full |
| No plateau and expiration < 15 s | Keep going until completely empty |
| FIVC > FVC | fill your lungs completely before blasting out |
| Suspected glottis closure | If you feel your throat closing, relax, but keep pushing |
| Suspected cough in first second of expiration | Try having a sip of water before the next blow |
| Suspected leak | Wrap the mouthpiece tightly in mouth during the whole maneuver |
| Suspected obstructed mouthpiece | Put tongue under the mouthpiece, relax |

Abbreviations see Table S1 legends.

**Table S4** Types of abnormalities and their respective prevalence

|  | **Prevalence** | | |
| --- | --- | --- | --- |
| **Types of abnormalities** | **Training set**  **(N = 14,124)** | **Internal test set**  **(N = 1,569)** | **External test set**  **(N = 182)** |
| **Visual** **abnormalities, n (%)** |  |  |  |
| Suspected glottis closure | 1,445 (10.2%) | 99 (6.3%) | 49 (26.9%) |
| Suspected cough | 663 (4.7%) | 2 (0.1%) | 3 (1.6%) |
| Suspected leak | 2 (0.0%) | 0 (0.0%) | 0 (0.0%) |
| Suspected obstructed mouthpiece | 229 (1.6%) | 41 (2.6%) | 47 (3.9%) |
| **Quantitative abnormalities, n (%)** |  |  |  |
| BEV exceeds limit | 461 (3.3%) | 4 (0.3%) | 30 (16.5%) |
| Rise time ≥150 ms | 516 (3.7%) | 42 (2.7%) | 7 (3.8%) |
| Unsatisfied EOFE | 374 (2.6%) | 32 (2.0%) | 8 (4.4%) |

Data are presented as absolute numbers in the case of frequencies. Abbreviations see Table S1 legends.

**Figure S1**

**Title:** Organizational structure of the cloud-based AI system.

**Legend:** The cloud-based AI system with three main roles: AI system, coordinator, and general practitioners. The study involved 10 selected PCi with 30 general practitioners. AI = artificial intelligence; PCi = primary care units.

**Figure S2**

**Title:** Example of the patient file with a complete spirometry test.

**Legend:** The spirometry file includes lung function parameters and curves is used in regular clinical practice in primary care. Example in the Chinese language.

**Figure S3**

**Title:** AI system used to evaluate each patient case.

**Legend:** The AI system assessed the quality of spirometry tests as the first object, with each maneuver being decided for FEV_1_ and FVC (decide separately) acceptability and usability, together with one of seven quality grades. If FEV_1_ and/or FVC were not acceptable/usable, then the warning messages and patient instructions would be described. FEV_1_ forced expiratory volume in 1s; BEV = back-extrapolated volume; FVC = forced vital capacity; AI = artificial intelligence.

**Figure S4**

**Title:** Method procedure.

**Legend:** The baseline performance of GPs was assessed in month 0 before intervention with the AI system. Then, GPs were instructed to manage the system after baseline data were collected on day 31, they were able to access the system during the period of the first and second intervention months (month 1 and month 2). GPs = general practitioners; AI = artificial intelligence.

**Figure S5**

**Title:** Proposed framework and Object Detection Module.

**Legend:** The framework mainly consists of three modules: Data Preprocessing Module, Rule Module, and Object Detection Module. Given input data, the Data Preprocessing Module will extract both numerical information and curve images. Then, the Rule Module will process the numerical information and output corresponding results (Yes/No). Simultaneously, the curve images are sent to the Object Detection Module which will automatically determine the type and location if an anomaly exists. Finally, the results of the Rule Module and Object Detection Module are combined together to generate FEV_1_ or FVC acceptability, usability, quality rating, and guidance for patients. RoI = region of interest; FEV_1_ = forced expiratory volume in 1s; FVC = forced vital capacity.

**Figure S6**

**Title:** Architecture of ResNet50-V1 backbone model.

**Legend:** Parameters of Convolution block, e.g., 1*1 conv 64: a convolution layer with a kernel size of 1*1 and 64 channels. Skip Connection: solid line denotes a skip connection without convolution operation, dotted line denotes a skip connection with additional convolution operation. Bottleneck: a unit architecture consists of 3 convolution layers, there are 16 bottlenecks in the network, we show 8 of them here for brevity. Maxpool /2: an operation that down-sample the input by a factor of 2.

**Figure S7**

**Title:** Confusion matrices of FEV_1_ and FVC acceptability and usability in the internal test set. 0: Not acceptable/usable, 1: Acceptable/usable.

**Legend:** a) Confusion matrix with counts of all correctly and incorrectly predicted FEV_1_ acceptable maneuvers in the internal test set; b) Confusion matrix with counts of all correctly and incorrectly predicted FEV_1_ usable maneuvers in the internal test set; c) Confusion matrix with counts of all correctly and incorrectly predicted FVC acceptable maneuvers in the internal test set; d) Confusion matrix with counts of all correctly and incorrectly predicted FVC usable maneuvers in the internal test set. FEV_1_ = forced expiratory volume in 1s; FVC = forced vital capacity.

**Figure S8**

**Title:** Confusion matrices of FEV_1_ and FVC quality rating (Internal/external).

**Legend:** a) Confusion matrix with counts of all correctly and incorrectly predicted FEV_1_ rating maneuvers in the internal test set; b) Confusion matrix with counts of all correctly and incorrectly predicted FVC rating maneuvers in the internal test set; c) Confusion matrix with counts of all correctly and incorrectly predicted FEV_1_ rating maneuvers in the external test set; d) Confusion matrix with counts of all correctly and incorrectly predicted FVC rating maneuvers in the external test set. FEV_1_ = forced expiratory volume in 1s; FVC = forced vital capacity.

**Figure S9**

**Title:** Confusion matrices of FEV_1_ and FVC acceptability and usability in the external test set. 0: Not acceptable/usable, 1: Acceptable/usable.

**Legend:** a) Confusion matrix with counts of all correctly and incorrectly predicted FEV_1_ acceptable maneuvers in the external test set; b) Confusion matrix with counts of all correctly and incorrectly predicted FEV_1_ usable maneuvers in the external test set; c) Confusion matrix with counts of all correctly and incorrectly predicted FVC acceptable maneuvers in the external test set; d) Confusion matrix with counts of all correctly and incorrectly predicted FVC usable maneuvers in the external test set. FEV_1_ = forced expiratory volume in 1s; FVC = forced vital capacity.
