# Supplementary figures and images for: Deep learning for spirometry quality assurance with spirometric indices and curves
Source: Respir Res. 2022 Apr 21;23:98. doi: 10.1186/s12931-022-02014-9 (PMC9028127; doi:10.1186/s12931-022-02014-9)

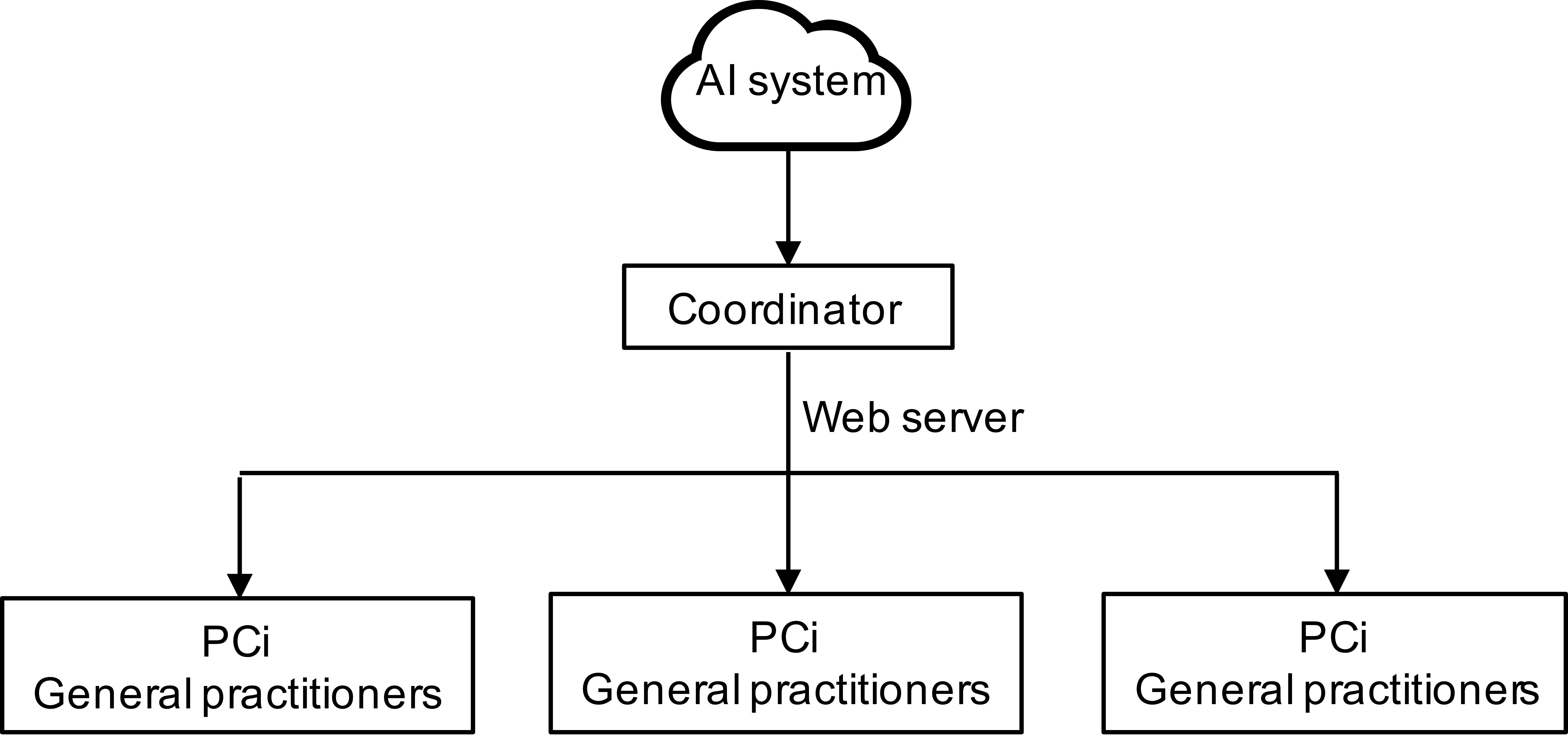

Supplement: Supplementary file 2 — Additional file 2: Fig. S1. Organizational structure of the cloud-based AI system. [file 12931_2022_2014_MOESM2_ESM.tif]

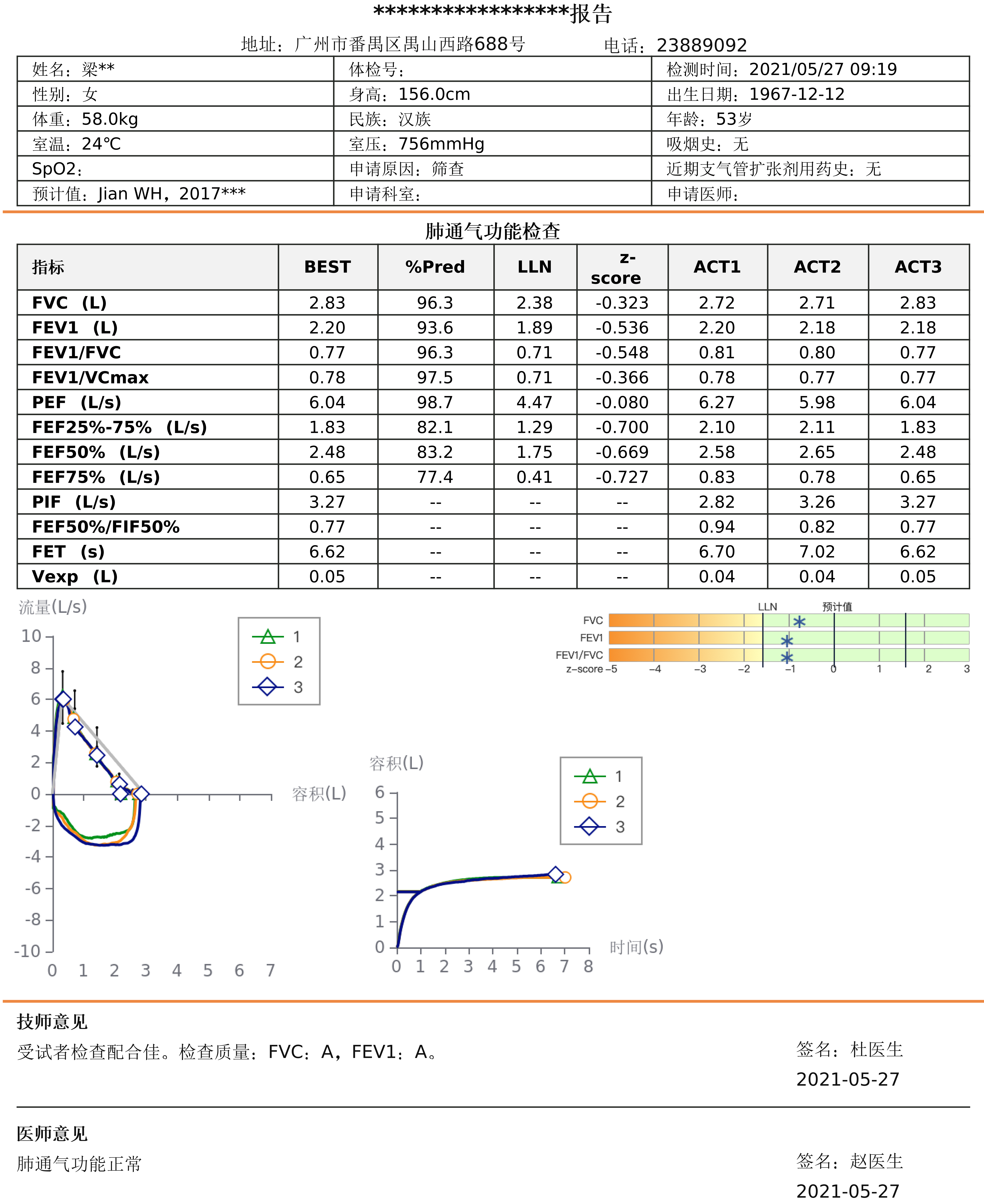

Supplement: Supplementary file 3 — Additional file 3: Fig. S2. Example of the patient file with a complete spirometry test. [file 12931_2022_2014_MOESM3_ESM.tif]

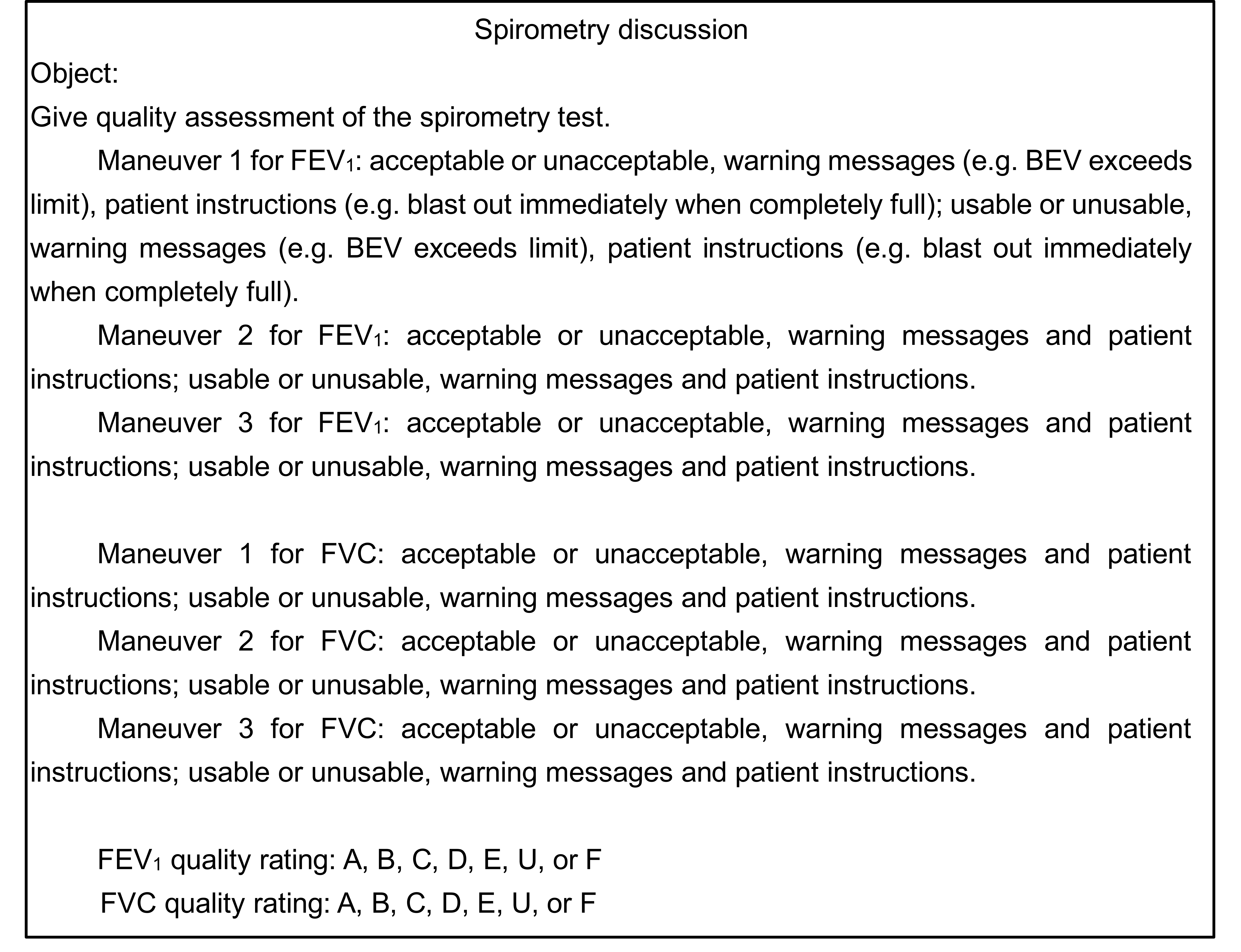

Supplement: Supplementary file 4 — Additional file 4: Fig. S3. AI system used to evaluate each patient case. [file 12931_2022_2014_MOESM4_ESM.tif]

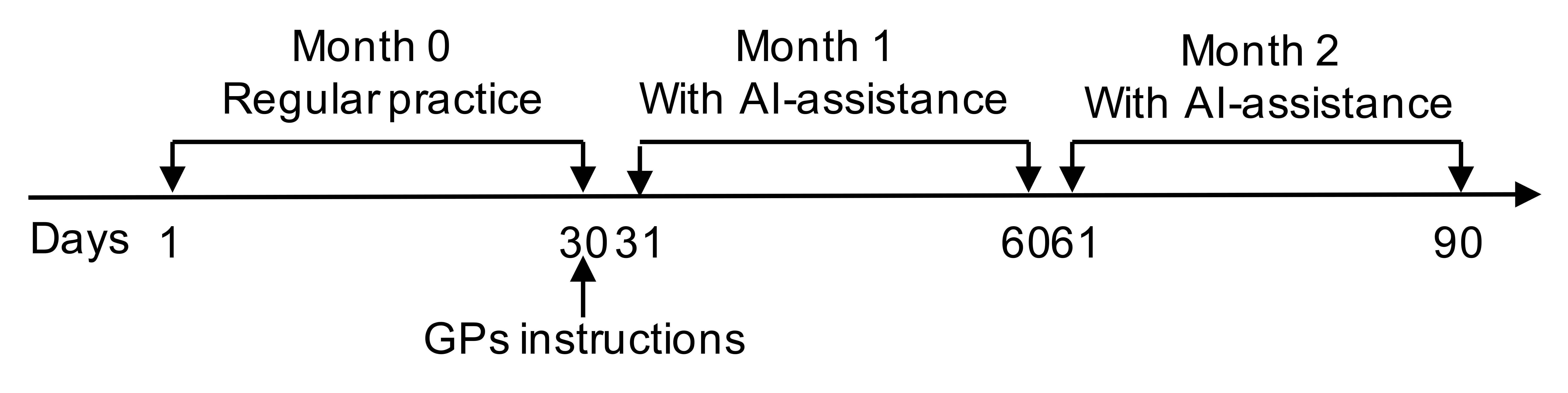

Supplement: Supplementary file 5 — Additional file 5: Fig. S4. Method procedure. [file 12931_2022_2014_MOESM5_ESM.tif]

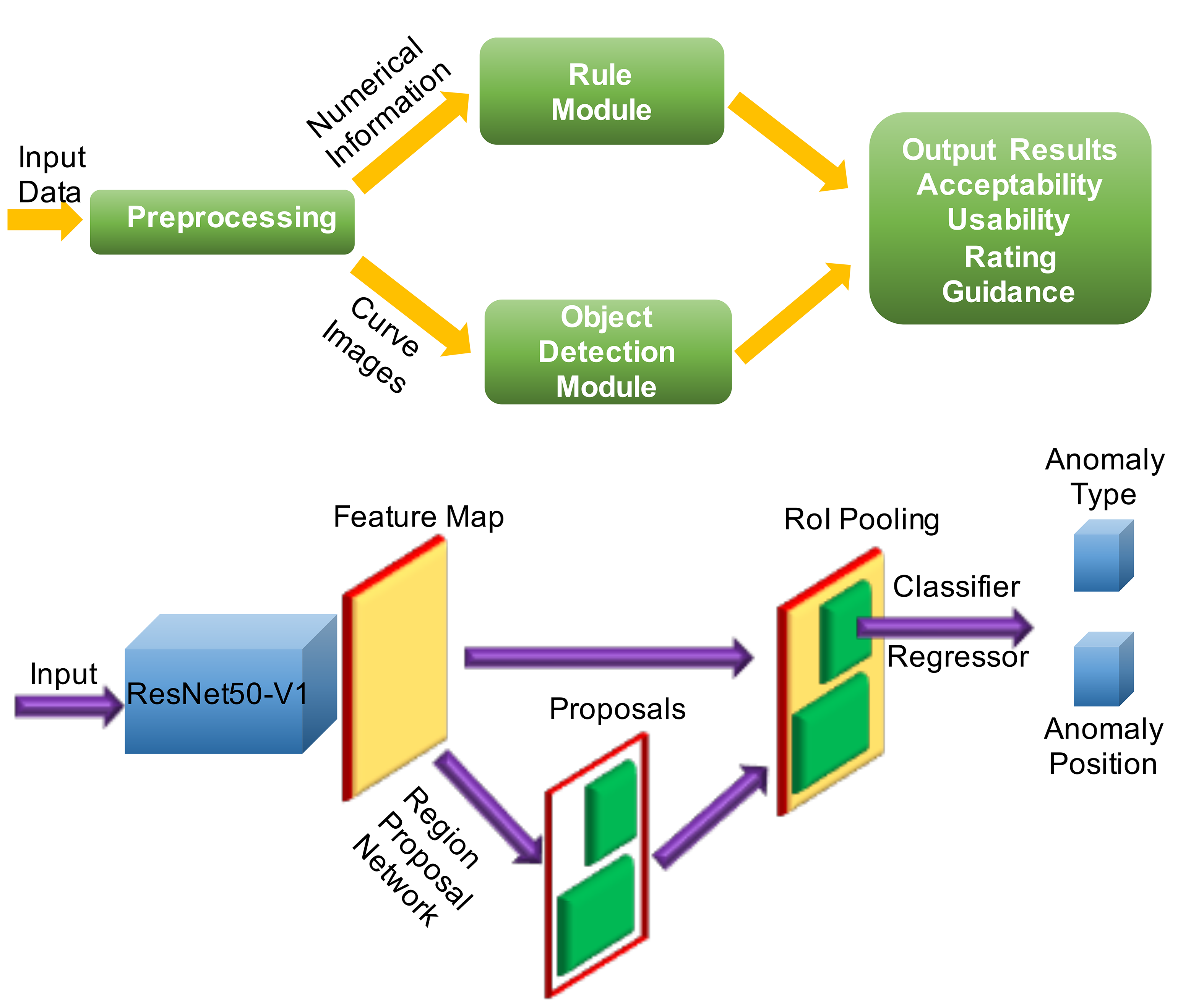

Supplement: Supplementary file 6 — Additional file 6: Fig. S5. Proposed framework and Object Detection Module. [file 12931_2022_2014_MOESM6_ESM.tif]

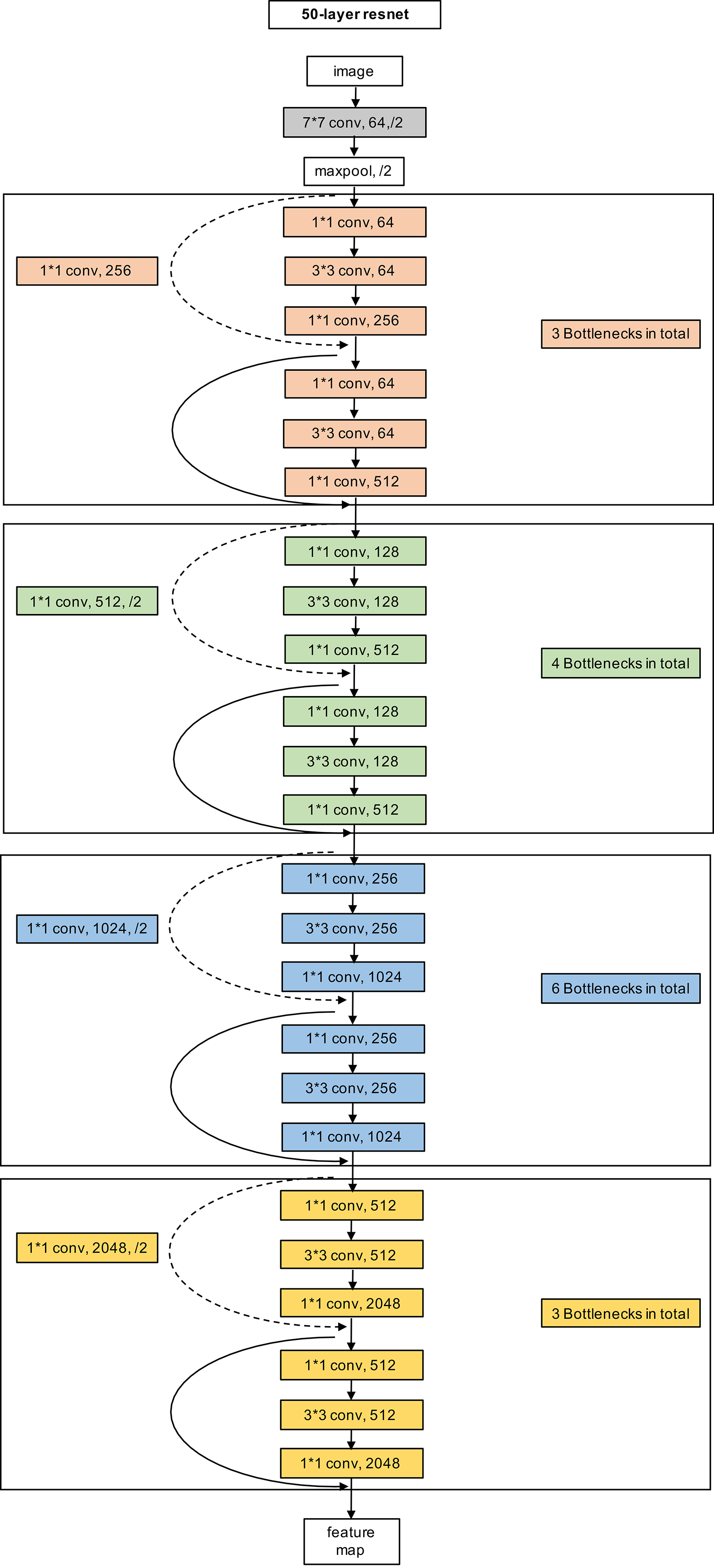

Supplement: Supplementary file 7 — Additional file 7: Fig. S6. Architecture of ResNet50-V1 backbone model. [file 12931_2022_2014_MOESM7_ESM.tif]

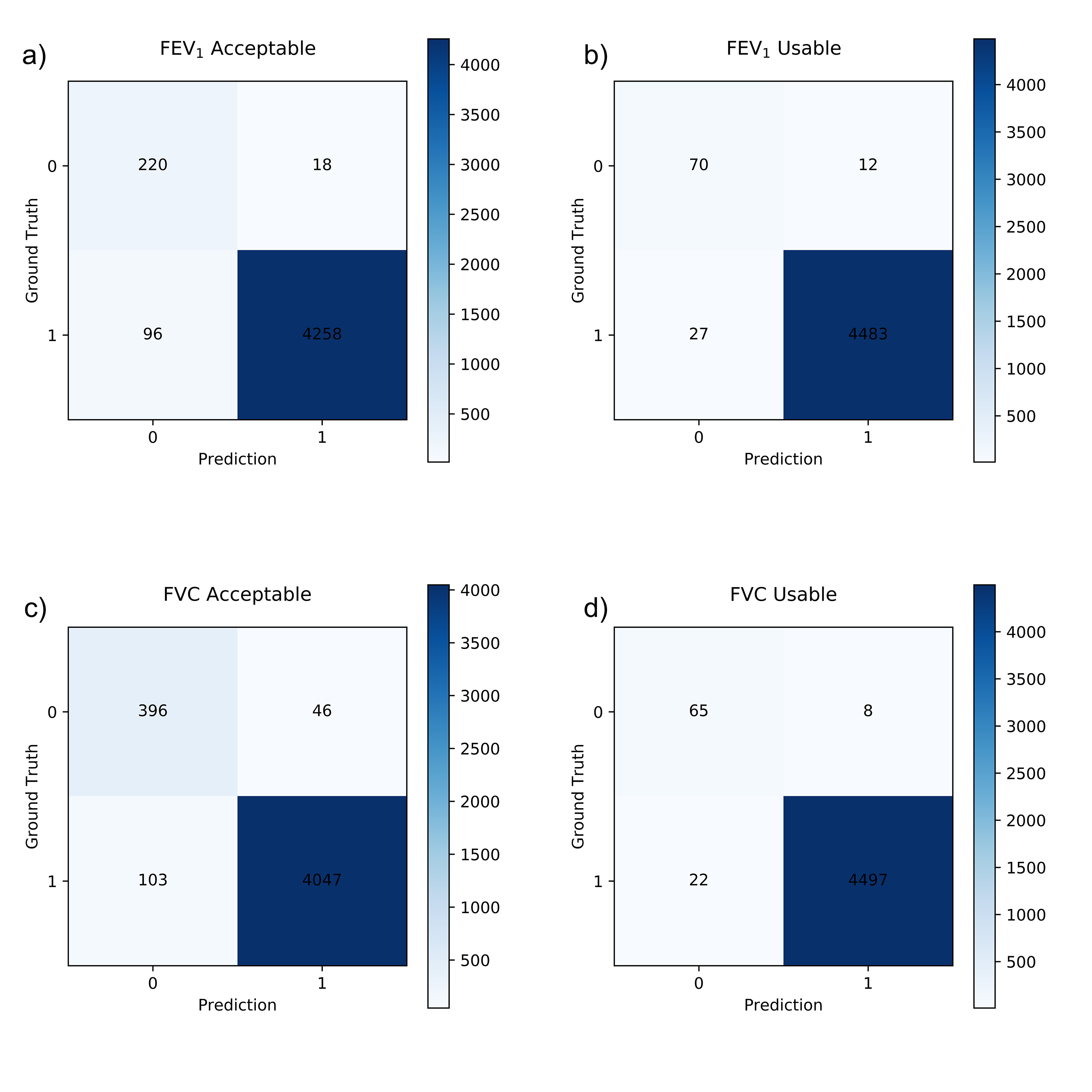

Supplement: Supplementary file 8 — Additional file 8: Fig. S7. Confusion matrices of FEV1 and FVC acceptability and usability in the internal test set. 0: Not acceptable/usable, 1: Acceptable/usable. [file 12931_2022_2014_MOESM8_ESM.tif]

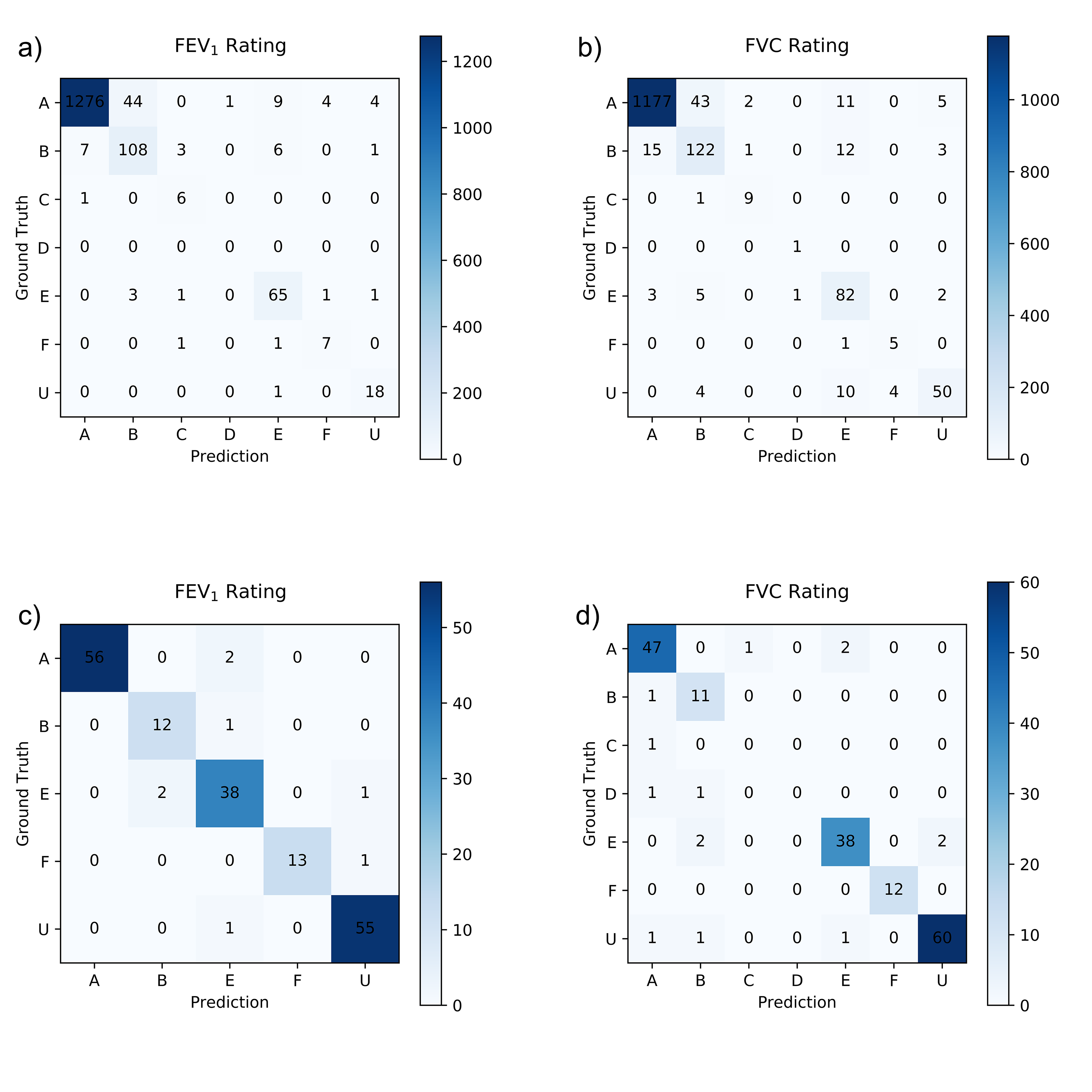

Supplement: Supplementary file 9 — Additional file 9: Fig. S8. Confusion matrices of FEV1 and FVC quality rating (Internal/external). [file 12931_2022_2014_MOESM9_ESM.tif]

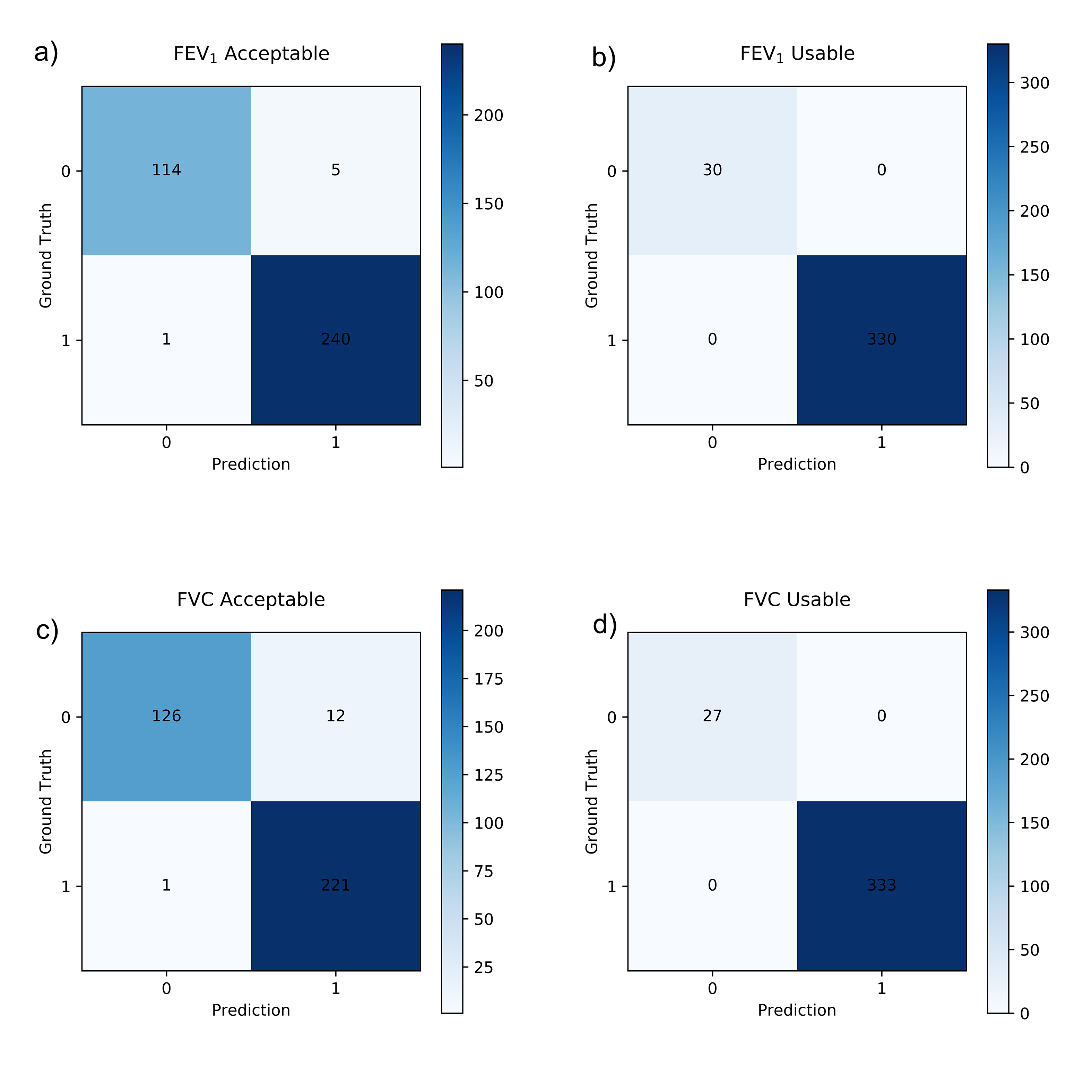

Supplement: Supplementary file 10 — Additional file 10: Fig. S9. Confusion matrices of FEV1 and FVC acceptability and usability in the external test set. 0: Not acceptable/usable, 1: Acceptable/usable. [file 12931_2022_2014_MOESM10_ESM.tif]
